# When Is a Medical Teacher or Practitioner Superannuated?

**Published:** 1857-04

**Authors:** 


### *When is a Medical Teacher or Practitioner Superannuated?*

In the organization of one of our western medical schools some years ago, the board of trustees of the institution declared, by the enactment of a statute, that when a professor has attained the age of sixty-five years he is no longer qualified to teach, but must consider himself superannuated, in spite of whatever testimony he may have to the contrary. In Paris, a law prevails in regard to hospital physicians and surgeons, similar in its bearing, but even still more arbitrary and unjust. since its limit is five years less. Under this regulation MM. Paul Dubois, Cruveilhier, Bonneau, and Hervez de Chégoin, having reached the fatal sixtieth year, retired from their respective posts at the beginning of the present year. M. Paul Dubois continues, however, as Professor of Clinical Midwifery to the Faculty at the Hôpital des Cliniques; it is at the Maternité that he will have a successor.—*Ib.*

